# Synchronous Nodal Involvement of Metastatic Adenocarcinoma and Classical Hodgkin’s Lymphoma

**DOI:** 10.4274/tjh.2016.0478

**Published:** 2017-08-02

**Authors:** Ritesh Sachdev, Shalini Goel, Ruchika K Goel, Smeeta Gajendra, Nitin Sood

**Affiliations:** 1 Medanta The Medicity Hospital, Lab Medicine and Transfusion Medicine, Departments of Pathology, Gurgaon, India; 2 Medanta The Medicity Hospital, Clinic of Medical Oncology and Hematology, Gurgaon, India

**Keywords:** Hodgkin’s lymphoma, Metastatic adenocarcinoma, Reed-Sternberg cells, PET/CT

## To The Editor,

The cervical lymph nodes (LNs), along with being the primary site of lymphomas, are also the draining sites for malignancies of the gastrointestinal tract, breasts, lungs, etc. Hodgkin’s disease also most commonly affects the cervical and axillary LNs. We, in the era of modern techniques for diagnosing malignancies, stress the fact that a diligent histopathological examination of the background lymphoid tissue is important to exclude a coexistent lymphoma, particularly after a metastasis is found.

**Figure 1:**
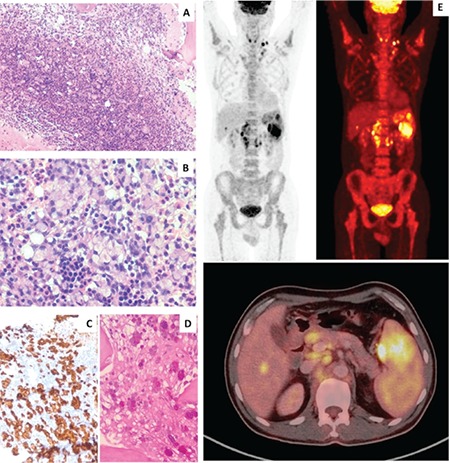
Bone marrow biopsy showing diffuse infiltration of the marrow spaces by acini and cords of signet ring cells with abundant mucinous cytoplasm (A, 100^x^; B, 400^x^); positive for cytokeratin immunostaining (C, 200^x^) and Alcian blue/periodic acid-Schiff (D, 200^x^). PET/CT revealed multiple fluorodeoxyglucose -avid hypermetabolic-enlarged lymph nodes with splenomegaly (E).

A 55-year-old hypertensive male presented with significant weight loss and vague epigastric fullness for the past month. On examination, splenomegaly (13.4 cm) and multiple left level IV and V cervical LNs measuring between 1 and 1.5 cm were noted. Investigations revealed anemia (73 g/L) with thrombocytopenia (120x10^9^/L). Peripheral smear showed a leukoerythroblastic blood picture, for which bone marrow (BM) examination was advised. The BM aspirate was hemodiluted; however, a BM biopsy showed diffuse infiltration by acini and cords of signet ring cells with abundant mucinous cytoplasm and eccentric nuclei (Figures 1A and 1B), which were positive for cytokeratin (Figure 1C, 200^x^) and Alcian blue/periodic acid-Schiff staining (Figure 1D). The surrounding area showed desmoplasia with normal marrow elements. Diagnosis of metastatic adenocarcinoma, possibly primarily from the gastrointestinal tract, was given and radiological studies were advised. Positron emission tomography-computed tomography revealed multiple hypermetabolic enlarged LNs in the left posterior cervical, level IV, supraclavicular region. The liver was enlarged with a small hypermetabolic hypodense area noted in segment VI. The spleen was enlarged with multiple hypermetabolic ill-defined areas (Figure 1E). Upper GI endoscopy showed scalloped folds in D2 with diverticula near the papillary opening. A duodenal biopsy was done, which confirmed the diagnosis of mucinous adenocarcinoma.

**Figure 2:**
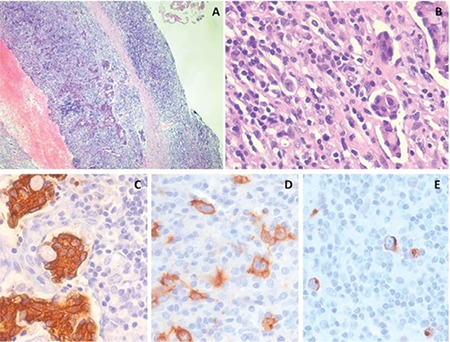
Histopathology of the lymph node showing complete effacement of lymph nodal architecture with presence of numerous Reed-Sternberg cells along with multiple intravascular tumor deposits (A, 100^x^; B, 400^x^). The intravascular tumor deposits were positive for cytokeratin immunostaining (C, 400^x^). The RS cells stained positive for CD30 (D, 400^x^) and CD15 (E, 400^x^).

Due to significant fluorodeoxyglucose (FDG)-avid lymphadenopathy, a left cervical excision biopsy was done. Histopathology of the LN revealed complete effacement of the lymph nodal architecture (Figures 2A and 2B) with thickened fibrous septa. The lymphoid nodules revealed the presence of numerous Reed-Sternberg (RS) cells surrounded by a monomorphic population of large cells with multiple intravascular tumor deposits predominantly in the periphery of the LN. The intravascular tumor deposits were positive for cytokeratin (Figure 2C). The RS cells stained positive for CD30 (Figure 2D) and CD15 (Figure 2E) and negative for CD20, CD3, and CD45. A diagnosis of a collision tumor comprising classical Hodgkin’s lymphoma and metastatic adenocarcinoma was made.

A collision tumor is defined as the concurrence of two histologically and topographically different neoplasms in one organ with little intermingling between the two neoplasms and without areas of transition between the abutting separate primaries. It occurs when two separate carcinomas metastasize to the same LN or when carcinoma metastasizes to the LNs that contain malignant lymphoma [1]. The etiopathology of these tumors is such that they are either a chance association or they share a common etiopathogenesis. It has been postulated previously that this can be the result of two simultaneously operating different processes, such as continuous proliferative stimulation and the presence of a common oncogenic factor such as Epstein-Barr virus [2]. Collision tumors with Hodgkin’s lymphoma as one component and solid organ malignancies as the other have been described in the literature, including metastases from breast and gastric carcinoma [1,3]. Synchronous tumors with Hodgkin’s lymphoma have been documented in the literature with medullary carcinoma of thyroid and colon carcinoma [4,5]. This case highlights the underlying synchronous nodal involvement of two divergent neoplastic populations. The need to look carefully at the background lymphoid population, particularly after a metastasis is found, is also emphasized.
